# Optimizing Sensor Position with Virtual Sensors in Human Activity Recognition System Design

**DOI:** 10.3390/s21206893

**Published:** 2021-10-18

**Authors:** Chengshuo Xia, Yuta Sugiura

**Affiliations:** 1 Graduate School of Science and Technology, Keio University, Yokohama 223-8522, Japan; 2 Faculty of Science and Technology, Keio University, Yokohama 223-8522, Japan; sugiura@keio.jp

**Keywords:** activity recognition, human–computer interaction, optimization, virtual sensor

## Abstract

Human activity recognition (HAR) systems combined with machine learning normally serve users based on a fixed sensor position interface. Variations in the installing position will alter the performance of the recognition and will require a new training dataset. Therefore, we need to understand the role of sensor position in HAR system design to optimize its effect. In this paper, we designed an optimization scheme with virtual sensor data for the HAR system. The system is able to generate the optimal sensor position from all possible locations under a given sensor number. Utilizing virtual sensor data, the training dataset can be accessed at low cost. The system can help the decision-making process of sensor position selection with great accuracy using feedback, as well as output the classifier at a lower cost than a conventional training model.

## 1. Introduction

The human activity recognition (HAR) technique is an effective tool for identifying human motions and has improved the daily lives of humans from multiple perspectives [1,2,3,4]. As a data-driven technique, regardless of the type of HAR system, the user or developer normally installs the sensor at the designated locations to obtain the signal for training [5,6]. The machine learning (ML) classifier was built based on such a position-specific dataset. Thus, the sensor position as an implicit interface can be recognized between the user and the built HAR system.

Currently, at the device level, the sensor adopted in the HAR system has been gradually combined with daily electronics, such as smartwatches and smartphones, and the user is required to wear the device or be active at specific locations. The inherent positioning of the HAR system enables the user to avoid selecting the sensor’s placement casually. However, fixed positions of employed equipment do not always work for all users. For the wearable type, the subject may have different body conditions and preferences for sensor wearing. Other locations combined with accessories like hair bands, headphones, braces, chest pockets, necklaces, knee/elbow supports, or shoes can also impact wearability [7]. For nonwearable types, the effective detection area is fixed by the installation of sensors. However, the indoor design of HAR systems generally takes into account plenty of potential locations to place the sensors. As different sensor positions will generate different data distributions which can affect the ML performance to a large extent, the selection of the sensor position is supposed to balance design convenience and system performance. Plenty of potential sensor positions exist in the design space so understanding the greatest accuracy of sensor positions and numbers is necessary for effective HAR systems. Therefore, from the point of view of human–computer interaction (HCI), providing a scheme to assist the sensor position decision-making process under a given condition benefits the HAR developers as well as the users.

Conventional HAR systems or sensor position related systems assumed a limited sensor position number. All considered sensor positions are supposed to place a real sensor and obtain the dataset. When the potential number of position increases, collecting all potential positions’ sensor data become unrealistic. Recently the technique of virtual sensors has emerged to reduce the position selection burden to an extent. Several researchers have started to create cross-modality virtual inertial measurement unit (IMU) sensor data to contribute to wearable HAR system design [8,9,10,11,12,13]. The use of virtual sensors can establish a training dataset in a relatively low cost and convenient way, and it is possible to overcome some obstacles that make real-world data difficult to collect. As an emerging technique, existing applications normally concentrate on augmenting the current dataset instead of a small scale related lightweight HAR system, such as focusing on a sensor position interface. The virtual sensor can draw back position selection for the user; however, there is still no system that focuses on exploring potential sensor positions to find the best position in an intelligent way. Although some works [14,15,16] appear to analyze the sensor position effect on HAR performance with a benchmark dataset, the benefits of these results are limited to the defined activity and classifier and are hard to apply to more generalized HAR systems. Particularly, there is no complete work to present the HAR system design dominated by the sensor position. Therefore, in this paper, we introduce the work of optimizing the sensor position combined with a virtual sensor to help intelligently design a lightweight HAR system. With virtual sensor utilization, we illustrate the HAR design within a small group of people as the case study.

The system is able to generate the optimal recommended positions (combinations) with a given sensor number. With virtual motion model input, the system can output the classifier under the optimal sensor position, which can be used in real-world recognition. The main contributions of this paper are as follows:

(1) The proposal of an optimizer to obtain the best sensor position during the HAR design process.
(2) The introduction of a generic virtual humanoid avatar-enabled simulated sensor design.
(3) An evaluation of a proposed optimization scheme with a real wearable case.
(4) A three-case study of the virtual avatar combined with optimization of the activity recognition system design.

## 2. Related Work

### 2.1. HAR with Related Sensor Positioning

With different devices used, each HAR system has advantages according to various applications. A camera as the most informative input source attracts close attention [5,6,17,18,19]. To apply the system in a pervasive way, a nonvision sensor, such as an inertial device, attached to the specific position of the body has also been widely used [15]. Additionally, some signals that can effectively reflect the variation of the body’s movement can also be adopted for motion estimation, such as the surface electrocardiogram signal of the forearm [20] and foot pressure [21]. A change of scenario can also be effectively adopted for motion recognition, such as using the infrared sensor to detect temperature changes [22], distance sensors for capturing the distance between the equipment and object [23], pressure-sensitive devices mounted on the floor, EEG signal [24], ambient light to estimate gestures [25], and utilization of Wi-Fi, RF signals [26], and so on.

From the point of view of wearable systems, Gemperle et al. [7] contributed work to define the wearability of the human body, including the possible position on a human body. Theoretically, unobtrusive placement on the body can be identified to place the sensor, which is mainly around the chief joint areas. However, regardless of the type of HAR system in a practical sense, the selection of sensor placement necessitates both consideration of space design and system performance.

### 2.2. Synthetic Sensor Data Based on 3D Virtual Motion

To facilitate the expansion of a nonvisual dataset for HAR, researchers are increasingly concentrating on generating simulated sensor data according to virtual human motion capture. Generally, the IMU data are synthesized. Most prior work has focused on using kinetics to calculate acceleration data from position variation [27,28]. IMUSim [9] first systematically introduced a method to generate synthetic acceleration, rotation velocity, and magnetic data with a Motion Capture (MoCap)-based human movement trajectory. This approach was mainly adopted for subsequent applications, for example, training the ML network from virtual avatar motion in Unity3D [10], gait analysis [29,30,31], and pedestrian trajectory [32]. To investigate motion with the fine-grained virtual sensor, Derungs et al. [29] attached plenty of virtual IMU sensors to a virtual avatar. However, they only considered gait motion, such as running, to analyze different sensor position effects on the gait marker parameters. Additionally, using the wider input source, some works began to use video as the data synthesis source [33,34]. Kwon et al. [8] designed the IMUTube to convert the RGB video into 3D virtual motion to generate acceleration data. In addition, Liu et al. [34] applied video information to extract the motion data of the finger to train the classifier via the computer vision model. Nevertheless, inferring the 3D joint movement trajectory from 2D RGB video still maintains inaccuracy, which is the bottleneck of applying such a method in dataset expansion.

Additionally, another scheme enabling the end-to-end ML network has also been designed [35,36,37]. Fortes et al. [35] designed the residual deep convolutional network, which is trained by the RGB video and related real IMU sensor data and can output the synthetic sensor data from a similar motion video. Alharbi et al. [36] developed the WGAN network to balance the data sample amount of the existing dataset.

### 2.3. Sensor’s Optimal Placement

As discussed, the location of the sensors can directly affect the result of the recognition system. To explore the positions with the highest accuracy, effective areas and potential positions can be optimized to find the best location. However, optimal sensor placement has seldom been studied in the HCI community. Kim et al. [38] conducted the first systematic examination regarding sensor placement for a computer mouse. In other fields, many researchers have studied the influence of sensor positioning from the perspectives of the human body [39] and structure monitoring [40]. For widely used wearable accelerometers, Kunze et al. [15] compared the positions of accelerometers placed on the body and proposed a method to eliminate the influence caused by various locations. Cleland et al. [16], Olgun et al. [41], and Gjoreski et al. [42] all tested sensors worn on the body with different positions and numbers. However, their work only offered the trend of accuracy, regarding disparate positions and numbers instead of answering where the best positions were for activity recognition. To optimize the sensor’s location for detection, it is more widely used in building/structure health monitoring. The sensor can be placed in an extensive space inside a building, like a living room. Using an optimization algorithm, such as a Bayesian network [43] or heuristic algorithm [14,44], has been identified as an effective tool for solving such problems.

## 3. Sensor Position Optimization in HAR Systems

### 3.1. Problem Description

**Figure 1:**
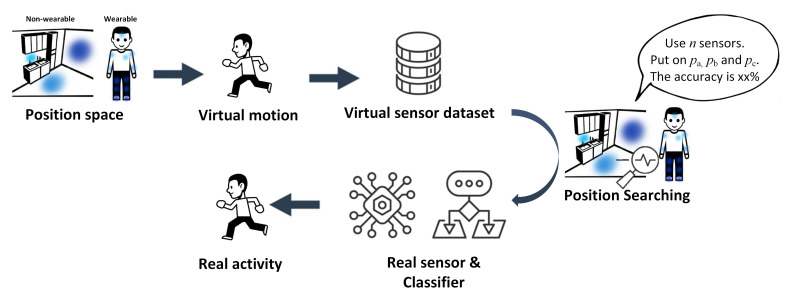
Overview pipeline of sensor position optimization with virtual sensor data.

For a common ML-based HAR system design, the developer normally follows the experience or trial-and-error approach to generate the dataset. Conventional methods can be recognized as the empirical method. Adhering to previous methods or experiences means the developer is unaware of how much accuracy improvement could exist if the sensor position changed or combined. To better support position selection, we focused on finding the best position of the sensor to derive the highest accuracy of HAR with a combination of different sensor numbers. It can be clarified as an optimization problem to derive the optimal solution that is able to generate a classifier with the best performance. Moreover, it is important to note that the optimal sensor position depends on each specific HAR system because a change in the recognized activity alters the corresponding optimal position as well. The pipeline is shown in Figure 1. The sensor position selection is supposed to be conducted during the design process, with the description of related variables as follows.

**Figure 2:**
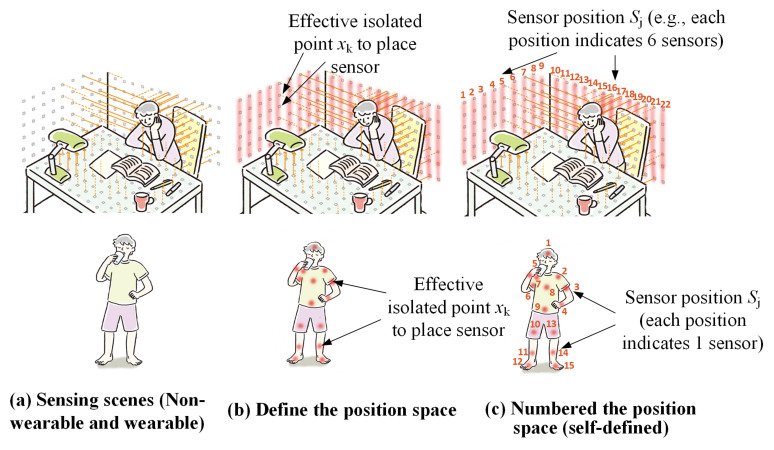
Sensor position illustration for different types of HAR system; (**a**) is the applied scene; (**b**) shows several effective isolated sensor points (such as the wall and significant joints of the body); and (**c**) is the predefined numbered position in the position space. For nonwearable types, one numbered sensor position can indicate several sensors (e.g., the figure shows six sensors at one position). For the wearable type, one sensor position indicates one sensor.

Sensor-position space: We define the *X* as any nonempty set that represents the sensor’s positions set. As the real sensor position is supposed to be a discrete point, the defined *X* is a discrete space. *X* = $\left\{x_{1},x_{2},\ldots ,x_{k}\right\}$, where *k* is the total number of effective isolated points in which a sensor can be placed. The nonempty subset $S_{j}$ ⊆ *X* represents sensor positions. The *j* indicates the number of defined sensor positions (e.g., *j* = 1, 2, …, 15 in wearable type and *j* = 1, 2, …, 22 in nonwearable type, as shown in Figure 2).

For the wearable type, the length of $S_{j}$ is normally one, that $S_{j}$ = $x_{i}$. The length of one means that only one sensor is placed at a time. For the nonwearable type, the length of $S_{j}$ can be one or more than one. For example, for densely placing sensors, we defined the length of $S_{j}$ as more than one, which indicates that multiple sensors are placed at a time, i.e, for one sensor position there is a series of sensors arranged. The illustration is given in Figure 2.

Optimized variable: Sensor position vector *p*, where *p* ∈$Z^{N}$. The *N* indicates the required sensor position amount (dimensions). $p^{I}$ is the sensor position equal to $S_{j}$ and *I*∈$\left\{1,2,\ldots N\right\}$.

**Figure 3:**
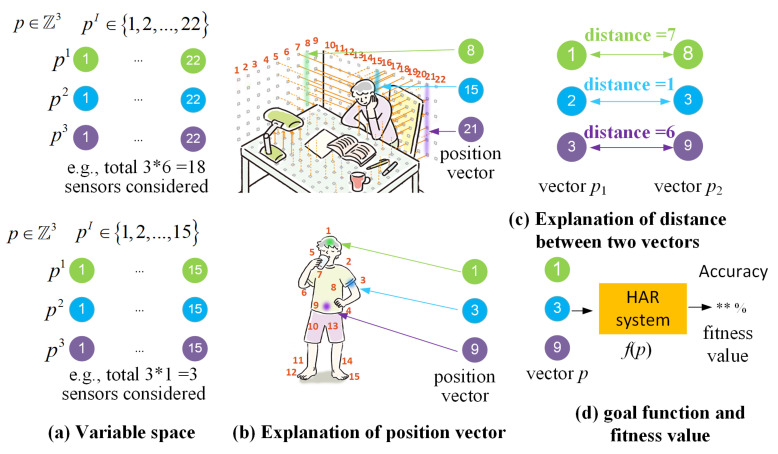
(**a**) is the variable space. For example, the position vector has three dimensions, i.e., *I* ∈ $\left\{1,2,3\right\}$. Each dimension $p^{I}$ is in $\left\{1,2,3\ldots ,22\right\}$ and $\left\{1,2,3\ldots ,15\right\}$ for nonwearable and wearable, respectively; (**b**) is the illustration of a specific sensor number corresponding to the real sensor position; (**c**) defines the distance of each dimension between the two vectors; and (**d**) is the definition of the goal function and fitness value.

Distance between position vectors: We define the distance between two sensor position vectors in each dimension. For example, for two vectors $p_{1}$ and $p_{2}$∈$Z^{3}$, the distance between two vectors is able to be $d_{j}$ = $p_{2}^{I}$−$p_{1}^{I}$ (as shown in Figure 3).

Goal function: *f*(*p*). The relationship between the sensor position and the accuracy of the built HAR system.

Fitness value: The value of *f*(*p*), i.e., the accuracy of the HAR system.

Best solution: $p_{best}$ which enables the maximum *f*(*p*) = *f*($p_{best}$).

### 3.2. Improved Discrete Cuckoo Searching (ID-CS) Algorithm

The mentioned sensor problem can be recognized as an NP-hard combination problem. The baseline method is dynamic programming, which tests all possible combinations of positions and outputs the best position with the maximum accuracy produced. Nevertheless, when the potential position and required sensor number are large, the baseline method will cause a curse of computational cost. A heuristic searching method provides the tool to produce the global optimal result with a reasonable time cost. In this paper, we utilized the cuckoo search (CS) optimization scheme with an improvement to enable it to be more suitable for the sensor position selection process [45]. A detailed introduction of the standard CS algorithm was introduced in [45]. CS is an efficient optimization tool, applied to multiple issues with different modes, but it is rarely used to optimize sensor placement [46,47,48].

CS utilizes Levy flights to seek a new nest for generating novel solutions and values for the objective function. The Levy flights are a type of random walk that obeys **Levy distribution**. It can effectively expand potential solutions and reduce the waste of computing resources in an uncertain space. Using the Levy flight strategy, the updating of each solution follows Equation (1) [45]:

(1) $$x_{i}^{n}\left(t+1\right)=x_{i}^{n}\left(t\right)\pm step=x_{i}^{n}\left(t\right)+\alpha ⊕\mathrm{Levy}\left(\beta \right)\left(x_{i}^{n}\left(t\right)-x_{best}^{n}\right)$$

where the *x* is the position of the nest and *i* is the iteration times. *N* represents the dimension, and *t* + 1 is the next new solution. The α is the step size and is applied to control the size of a position change for each iteration. ⊕ indicates multiplication; $x_{best}^{n}$ represents the current best solution. It is supposed to adapt the optimization task to enable as many solutions as possible and also ensure the speed of convergence. The Levy flights are calculated from the Levy distribution given in Equations (2)–(4) [45].

(2) $$\mathrm{Levy}\left(\beta \right)∼\frac{\mu }{{\left|v\right|}^{\frac{1}{\beta }}}$$

(3) $$\mu ∼N\left(0,\sigma_{\mu }^{2}\right),\;v∼N\left(0,\sigma_{v}^{2}\right)$$

(4) $$\sigma_{\mu }={\left\{\frac{\Gamma (1+\beta )\sin(\pi \beta /2)}{\Gamma \left[\left(1+\beta \right)/2\right]\beta 2^{(\beta -1)/2}}\right\}}^{1/\beta },\sigma_{v}=1$$

where two significant parameters μ and *v* follow the normal distribution and $\Gamma \left(x\right)=\int_{0}^{\infty }e^{-t}t^{x-1}dt$ is the Gamma function. The nest’s position should be an integer. Several studies have applied CS to the discrete problem [46,48]. However, for addressing the sensor placement problem, one of the most significant parts is to avoid the repetition of each sensor’s position in one dimension. The options for the sensor’s placement are limited, which gives a boundary during optimization. We reference the method of [14] to avoid position repetition and boundary limitations during the iteration.

The process of optimization using CS can be viewed as a Markov process in which the current change in position relies only on the previous state. The key point is how to control the random walk, the Levy flight, so that solutions can move to the best solution more quickly instead of wandering away from the potential best solution. Therefore, we mapped the Levy distribution into a specific range via the **tanh ( )** function (as shown in Equation (6); the value range of the standard tanh( ) is [−1,1]). We then discrete each value of a transformed Levy flight. Each number of interval divisions is used as a step length (i.e., the 1, 2, …, γ).

(5) $$s=\alpha ⊕\mathrm{Levy}\left(\beta \right)\left(x_{i}^{n}\left(t\right)-x_{best}^{n}\right)$$

(6) $$s^{\prime }=\gamma \tanh\left(s\right)=\gamma \frac{e^{s}-e^{-s}}{e^{s}+e^{-s}}$$

(7) $$step=\left\{\begin{array}{cc} 1 & \;\mathrm{if}\;s^{\prime }\in \left(-1,1\right)\\ 2 & \;\mathrm{if}\;s^{\prime }\in \left(-2,-1\right]\cup \left[1,2\right)\\ \ldots & \\ \gamma & \;\mathrm{if}\;s^{\prime }\in \left(-\gamma ,-\gamma +1\right]\cup \left[\gamma -1,\gamma \right) \end{array}\right.$$

**Figure 4:**
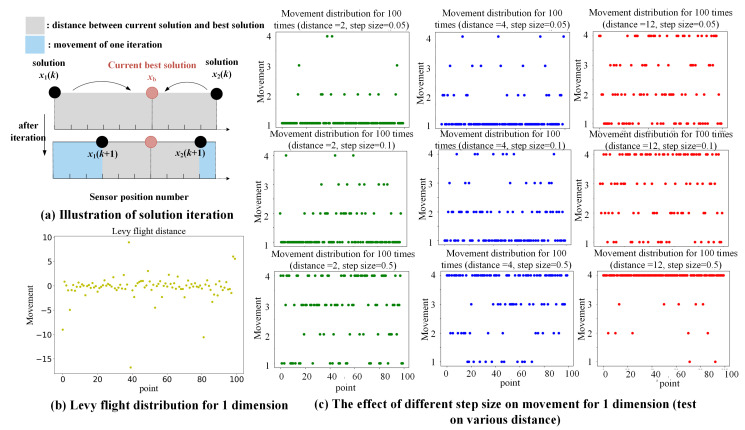
The distribution of adjustment of the solution regarding the scale factor in 100 iterations (the mapping operator is 4 as an example); (**a**) Illustration of the distance between the solution and the current best result, as well as the adjustment of each iteration; (**b**) the initial Levy flights distribution for adjustment during 100 times iteration; and (**c**) the influence of step size on different distance situations.

**Figure 5:**
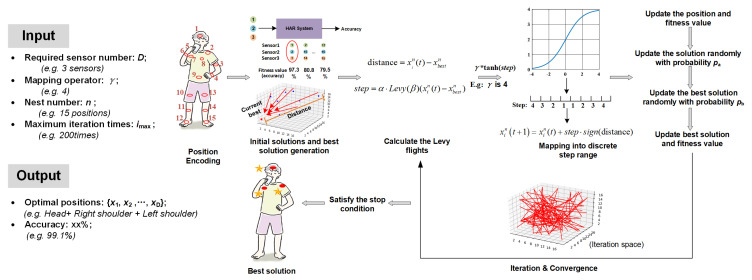
Working process of the proposed ID-CS optimization. After the position space has been defined, the initial solutions and the initial best solution are generated. Then, the iteration starts. The distance between the best solution and the current solution is obtained and multiplied by the mapped Levy length with a step-scaling factor γ to decide the movement of the solution. With probability $p_{a}\in \left(0,1\right)$ the current solution will be changed randomly to a new one. Moreover, to help the best solution escape the local optimum trap, the fraction could disturb the position of the existing best solution. Finally, we compared each fitness value to update the best solution until the stop condition is satisfied.

Here γ is the element enabling the mapped range to [−γ, γ]; we call it the “mapping operator”. It determines the maximum movement of each iteration. In addition, *s* is the length of the Levy step. The step is the ultimate length of the position adjustment. As shown in Figure 4, the initial Levy flight distributes randomly. Figure 4c gives a series distribution figure regarding the different distances and the step size, α. It can be seen that the value of the step size determines the adjustment of position to a large extent. A large value of α will cause more adjustment, which means the convergence speed will be improved while fewer solutions are able to be tested. For longer distances, a larger α value is able to fasten the convergence speed, while for shorter distances, it will lead to missing more possible solutions. On the contrary, a smaller α can help the iteration to test more solutions, but the speed will be scarified. Finding a favorable value of α is helpful to balance the convergence speed and to test more solutions. In this paper, we selected the α as 0.1. The entire process of the proposed ID-CS optimization is shown in Figure 5.

## 4. Virtual Sensor Design Based on Humanoid Avatar

As introduced in Section 2.2, to optimize the potential sensor position space and decrease the burden of training real dataset collection, the virtual sensor design can be utilized. Thus, it is suitable to be selected as a tool to apply the proposed optimization scheme and to form a novel design pipeline for small scale HAR systems in a lightweight and convenient fashion.

### 4.1. Virtual Activity and Environment

To obtain the virtual sensor signal, generally, the virtual activity is required to be played in a virtual 3D environment as motion in the real world at first. The method to obtain the virtual activity can be divided into three types: self-generated avatar, predefined animation avatar, and RGB video source based. The self-generated avatar requests that the user conducts the activity in the real world at first and uses the motion modeling tool to reconstruct the motion trajectory in the 3D virtual environment (like the off-the-shelf MoCap device, Kinect, and Xsens). The predefined animation avatar is the existing animation data that help the designer in game development and aim to produce a realistic action, such as Adobe Mixamo. Video source-based technology relies on computer vision and deep networks, and the depth of the 2D figure can be estimated and used to transfer the 3D local position data into a 3D global position [8].

The self-generated avatar is the most accurate way to gather the arbitrary virtual motion model, while its shortcoming is obvious: it requires the user to wear the MoCap device or conduct the activity before the depth camera. The pre-animation avatar decreases the matter of the user, but the category of virtual activity and length of activity (normally a few seconds) both limit this method. Finally, the video-based method creates a great opportunity to collect multiple virtual activities. However, because the 2D video lacks depth information, the estimation of 3D reconstruction still faces inaccuracies, especially when it is applied to generating other modality sensory data.

**Figure 6:**
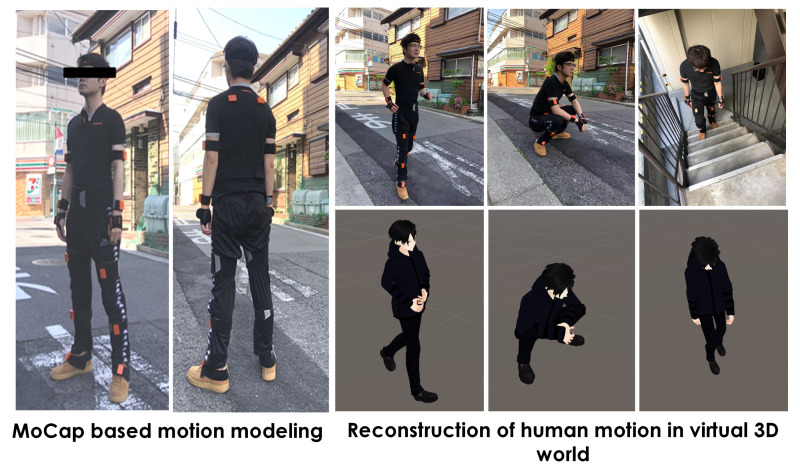
The 3D virtual human reconstruction. The subject is requested to wear the off-the-shelf MoCap suit (Xsens MVN). The related “.fbx” file can be converted and then applied to the virtual humanoid avatar to reconstruct the motion process. (The figures only present one frame of the whole process so there may be some inconsistency between the real and virtual motion).

In this paper, we utilized the virtual sensor to generate the signal data of different positions without real-world collection and aimed to validate the designed optimizer of the sensor position. Thus, we chose to use the self-generated avatar to define the customized activity from the subject and exploit the MoCap device to model the virtual activity in a game development environment—Unity3D (as shown in Figure 6).

### 4.2. Virtual Sensor Data Generation

The reconstructed motion is normally converted to a series of skeleton position data in frame-by-frame form (e.g., .fbx or c3d files). Thus, the acceleration and rotation data can be obtained through the second derivation of position data [9]. To obtain the distance data, we utilized the rigid body of the applied humanoid model to realize distance detection between the signal transmitter and the virtual human body part. For distance sampling, the Raycast function is utilized to simulate the infrared signal which can return the distance between the obstacle and the emission source.

**Figure 7:**
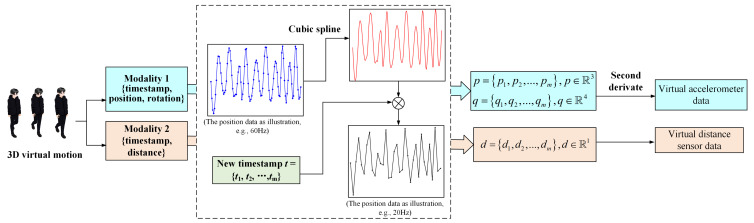
Virtual sensor implementation flow. The virtual data are taken together, along with time stamps. As the sampling time interval was not stable at this time, we exploited the cubic spline to reconstruct the continuous function between the time and the sampled signal value. Compared to the actual sensor system’s sampling characteristics, the virtual sampling frame rate can be adjusted by redividing the time domain with a new time interval. It therefore realizes arbitrary frame rate sampling and can correspond to the real sensor.

Replaying the virtual activity in a 3D environment to calculate virtual sensor data is recognized as a resampling process. The data type is converted from discrete to discrete. Thus, the time interval between each frame (i.e., the sampling frequency) would not always be the same and would be affected to a large extent by the situation of the device. Therefore, we defined the framework to generate the simulation signal from the 3D environment (as shown in Figure 7. The *p* and *q* represent the position and quaternion of body parts, respectively [9]. *d* indicates the distance).

One still needs to be aware that the virtual sensor data generation defined in this paper does not consider the noise effect. Although the high frequency noise most appeared during the real measurement, the noisy signal was normally eliminated by a low-pass Butterworth filter or another powerful filter. As we do not need to compare the sensor level signal between the virtual data and the real data, we may assume that the virtual signal is the filtered signal and idealized, and also applies the filter with real sensor data.

### 4.3. Virtual Data Augmentation

To improve the ability to classify data sensed in the real world, data augmentation (DA) was adopted to expand the dataset from individuals. Referencing the work of [49], the DA for wearable sensor data can be performed in the original dataset or feature domain. The sampled data are supposed to be represented by temporal and magnitude. Thus, the selected DA methods are related to the time and magnitude of sampled data. We adopted the approach described as follows:

- Permutation: Perturb the time location of input data. In a single data window, the data is divided into several segments and then randomly permuted.
- Time warping: Distort temporal locations. Similar as before, the several divided segments from the initial window are re-arranged by different time locations.
- Magnitude warping: Warp the signal’s magnitude. The magnitude of the data is convolved by a smooth curve varying around one.

However, for nonwearable types, HAR is performed based on the grayscale figure, which we select to change the humanoid’s size, especially the shoulder and chest width, to simulate the different body types below.

- Change the size of the model: Multiply by a factor of 1.2 and 0.8 to alter body type to simulate different body shapes.

## 5. Experiment and Results

### 5.1. Introduction to Evaluation Process

In this section, we introduce experiments regarding the optimization of the sensor position in HAR design. Two experiments are introduced below.

(a) Evaluation of the proposed optimization method.
(b) Case study showing the sensor optimization method to help develop several types of HAR systems.

We conducted our system on a laptop (ThinkPad, X1 Carbon; i7-8565U). The virtual human motion is generated from the commercial motion tracking product, Xsens. Virtual data acquisition is completed in Unity3D (14 April 2018), and data processing is realized in Python (Python 3.7). The same data-processing method was exploited for both actual sensor data and virtual sensor data. From the data generation section, we requested that subjects wearing the Xsens sensor perform the required activity repeatedly in 90 s as a data source. The generated virtual motion will be played in a 3D environment for the same length of time as in the real world. The sampled sensor data is augmented and subsequently segmented by a 2 s window (which can cover a single motion).

As a conventional handcrafted feature-based model structure still reveals superiority in HAR, especially when the training dataset is not large, we selected the support vector machine (SVM) model (with an RBF kernel, *C* = 1000) as the classifier [50]. Regarding extracted features, for a wearable case, we adopted the time and frequency features described in [14]. Calculated features are subsequently normalized to eliminate the effects of the amplitudes of different input signals. For the nonwearable case, we normalized the distance value into 0–255 and converted the signal from a specific position into a pixel of a grayscale figure. The figure width and length are related to the used numbers of sensors and the window size, respectively. The texture feature is extracted via the Gabor filter and input into the classifier.

To obtain the optimal sensor position in the virtual environment (Study 2), five-fold cross validation is first used in the virtual dataset to calculate the accuracy as the value of the goal function for optimizing. The found optimal position-based dataset is supposed to have the highest accuracy in a virtual dataset. When testing the built classifier against real sensor data, the whole virtual dataset is used for training, and the real dataset is used for testing.

### 5.2. Study 1: Implementing the ID-CS on Real Wearable Acceleration Sensor Data

Purpose: To test the performance of the developed optimization method with a benchmark dataset (real dataset).

Experiment configuration: To test the algorithm, the experiment can be scripted as follows:

(1) Objective: Find the optimal sensor combinations on the body;
(2) Sensor number: 3;
(3) Potential positions: 17 parts on the human body—head, chest, waist, right upper arm, right forearm, right hand, left upper arm, left forearm, left hand, right upper leg, right lower leg, right foot, left upper leg, left lower leg, left foot, left shoulder, and right shoulder;
(4) Recognized activity: standing/walking/running/sit-to-stand/stand-to-sit/squat-to-stand/stand-to-squat/upstairs/downstairs;
(5) Data: Acceleration data from 10 people (five females and five males; average age: 24). Subjects were asked to perform the above activity in 90 s with the Xsens device (60 Hz).

For comparison, the standard discrete cuckoo search (DCS) [45] with no interval division mapping, other swarm intelligence algorithms, the Discrete Firefly algorithm (DFA) [51], and Discrete Particle Swarm Optimization (DPSO) [52] were utilized to solve the same optimization problem. Additionally, Xia et al. [14] proposed an improved optimization scheme based on the DPSO algorithm to find the best sensor position on the human body. We also selected this method as a comparison. The same method of discretizing the iteration particle’s position value was applied to all swarm-based optimization algorithms.

For the baseline method, we first selected the empirical method from the work [53], which gives the statistical results of wearable accelerometers’ locations regarding different sensor numbers. Secondly, the random selection randomly generates sensor numbers in 200 iteration times. To show the best global solution, we also conducted the dynamic programming method that tests all the possible combinations to calculate the global optimal result.

These methods, except for the empirical and dynamic programming methods, were tested under the same iteration number (200 times). In this section, the entire real sensor data were employed to test the performance of different optimization methods. The collected real dataset was randomly divided into a training set and testing set with 60% and 40% of the original dataset, respectively. The accuracy of HAR is therefore calculated by the prediction rate from the testing set. As more practical sensor systems currently operate with a lower sampling rate to extend the work life of the battery, we also down-sampled the original dataset to 20 Hz and applied the same splitting method. Thus, a total of two datasets are employed to evaluate the methods.

Result: The results are given in Table 1. From the results, the designed ID-CS algorithm showed the best performance among the different swarm-based intelligence optimization algorithms. To validate how close the ID-CS found by our optimizer was to the best true global solution, we used the dynamic programming method to test all combinations. The results from both datasets show that the ID-CS not only outperforms other algorithms under the same iteration times but is also the closest to the global optimal solution with fewer computation times.

### 5.3. Study 2: Implementing the ID-CS on Virtual Sensor Data in HAR Design

Purpose: In this study, we aimed to show cases exploiting optimization combined with virtual sensors to design a HAR system, demonstrate the possible application, and further evaluate the effectiveness of the proposed optimization method. The study was conducted in the context of a nonwearable and wearable HAR system. Both on-body and environmental sensors were involved.

Experiment configuration: We invited nine participants to take part in the experiments and proposed three cases to apply the developed position optimization scheme with virtual sensors to design a real HAR system for a small group of people. The subjects were requested to wear the Xsens sensors to generate the virtual motion. We also provided a comparison in terms of the sensor selection method in ID-CS, the empirical method, the random selection method, and the dynamic programming method, to show how the sensor position selection method affects the final HAR accuracy.

(a) Case 1: Wearable accelerometer HAR system

**Figure 8:**
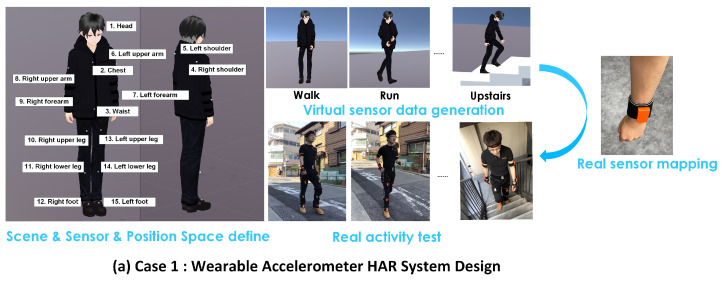
Case 1: Wearable accelerometer study; 15 sensor position spaces are found, 5 activities are recognized.

In this case, two males and one female (age: 21/26/24) were recruited. The data frame rate was 60 Hz and a total of 15 positions of body parts were considered. The mapping operator and nest number of ID-CS were set as 3 and 15, respectively. As the MoCap system was established based on the IMU device, real sensor data were adopted from MoCap’s sensor itself. The related process is shown in Figure 8.

(b) Case 2: Wearable distance sensor HAR system

**Figure 9:**
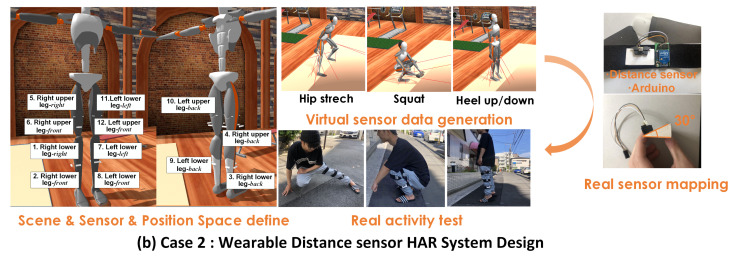
Case 2: Wearable distance sensor study; 12 sensor position spaces on the lower body are defined, 3 exercises are recognized.

In this case, the distance sensor is considered another sensor modality for recognizing exercises (as shown in Figure 9). The sensors attached to each body part have multiple directions and transmission angles. We normally supposed three directions—anterior, posterior, and outer—for each part. Thus, 12 positions from the lower limbs are provided. Three people (males; age: 19/26/27) are invited to test this application. Three types of exercises, the “heel up/down”, “squat”, and ”hip stretch”, are proposed as recognized activities. The transmission angle was set at 30 degrees. We mapped the distance scale in the real world into a virtual environment by calculating the rate between the humanoid model height and the real subject’s height. The data frame rate was 50 Hz and the mapping operator and nest number of ID-CS were set as 3 and 12, respectively. Regarding the empirical method, which is different from the large investigation of wearable IMU systems, designing a new type of such wearable distance system lacks the benefit of previous experience. We therefore asked the participants about their preferred position in the 12 involved positions. The preferred positions were selected as the tested objects for the empirical method.

To build the prototype of the distance sensor based HAR, we adopted the infrared distance sensor (GP2Y0A21YK0F, Sharp), which has an effective detection range from 10 to 80 cm. Additionally, the Arduino chip (ARDUINO PRO MINI) is used for data acquisition and transmission. Each subject must execute activities lasting for 60 s with a 50 Hz sampling rate for real-world testing.

(c) Case 3: Nonwearable distance sensor HAR system

**Figure 10:**
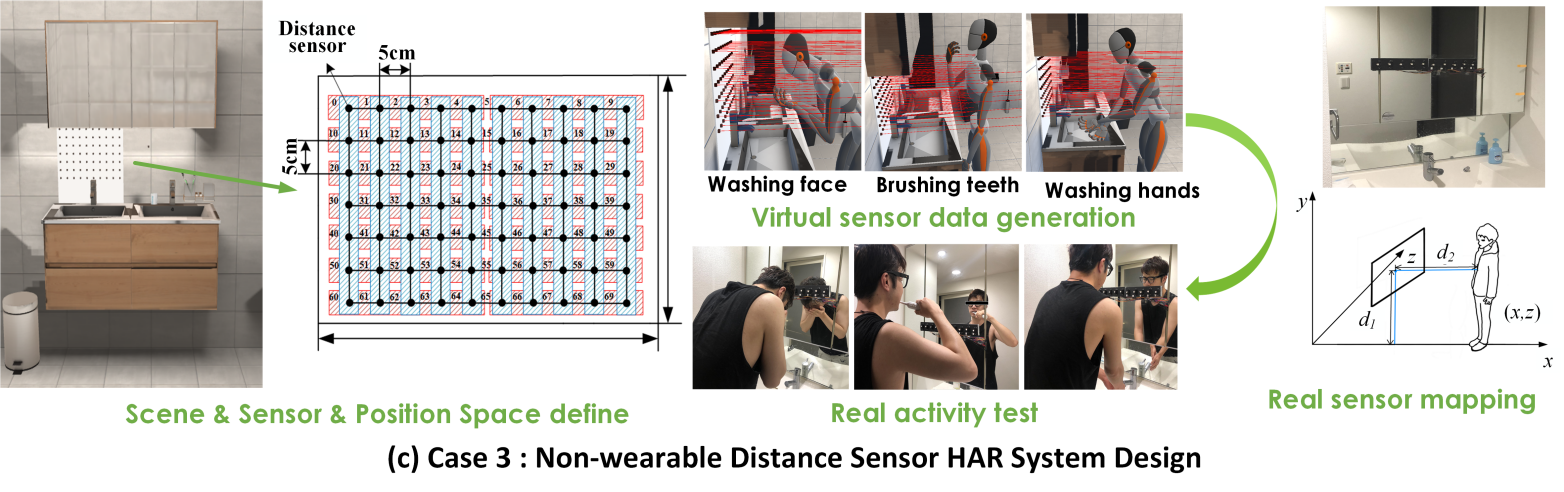
Case 3: Nonwearable distance sensor study. The applied scene is a bathroom. A total of 24 sensor position spaces are found (as the blue/red sensor board position shows) to recognize three types of daily activity.

The distance sensors were exploited as the environmental sensors in this case to detect the daily activities. The applied scene is selected as a bathroom, and activities like “washing hands,” “washing face,” and “brushing teeth” can be recognized. Three subjects (males, average height: 175, average weight: 65 kg, average age: 23.3) who lived together were invited to test the experiment. For this type of application, the optimized object will find the installing positions of the sensors under the preferred sensor number. We assumed a 7*10-sensor mounted on the wall above the washbasin (as shown in Figure 10 as the original area). A total of 70 sensors were used, and the interval between each sensor was 5 cm. We arranged the 70 sensors into 24 sub-boards. The first type contained five sensors, and the second had seven (cf. the blue and red blocks in Figure 10). Considering the real sensor system characteristics, we applied a 25 Hz sampling rate. As 24 sub-boards were defined, the total sensor possible positions were 24. The mapping operator was set as 4, and the nest number used in optimization was 20.

In this case, we did not conduct the so-called empirical method because the applied object was not related to the wearability. Different from the limited human body segments, the ambient area normally has enormous locations, and it is hard to define the empirical positions. Thus, we compared ID-CS with random selection and dynamic programming.

We utilized the infrared distance sensor (GP2Y0A21YK0F, Sharp) and the Arduino chip (ARDUINO PRO MINI) for data sampling and validation. The built sub-sensor board is shown in Figure 10. Real data sampling (25 Hz) with real sensors lasted for 60 s. We used the scale factor to map the subject’s coordinate in the virtual to real world. As shown in Figure 10, two important coordinates, ($x_{s}$,$y_{s}$,$z_{s}$) and ($x_{h}$,0,$z_{h}$), are required to reflect the real position of the sensor boards and the subject’s position to the virtual environment, respectively. The user is asked to complete the three types of activities in front of the sensor boards.

**Figure 11:**
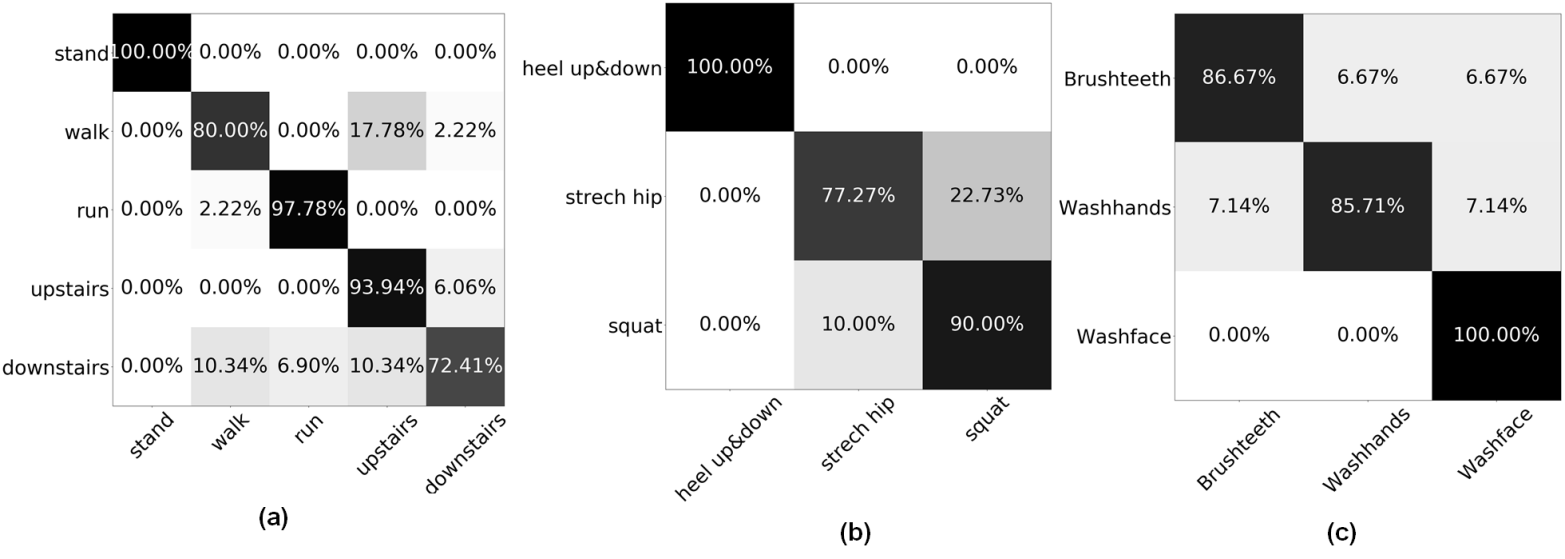
The confusion matrix of using a virtual classifier to recognize the real activity; (**a**) the results of Case 1; used positions are chest, right lower leg, and left lower leg; (**b**) the results of Case 2; the sensor positions are left lower leg-left and left upper leg-left; and (**c**) the results of Case 3; two sensor boards are used, i.e., the location number of [0, 1, 2, 3, 4] and [5, 6, 7, 8, 9].

Result: Table 2 and Table 3 present the results of the three-case evaluations of different types of HAR systems in terms of the sensor position selection. Different methods are exploited to find the sensor position with the related HAR system’s accuracy. After the sensor position based virtual HAR systems have been established, the trained classifiers are used to recognize the real activities. Figure 11 presents the confusion matrix result of using a virtual classifier to recognize real activities, which expresses the effectiveness of such a classifier trained by virtual sensory for real-world application. The corresponding accuracy of using the founded sensor position dataset are 89.85%, 91.25% and 90.69% with Case 1, 2, and 3, respectively.

## 6. Discussion

### 6.1. Optimization of the Position Space

The selection of the sensor position is dependent on the defined position space, which is determined by the required application. The wearable HAR system can normally consider the whole body or specific limbs as the position space. For nonwearable types, especially for a densely placed scene, the position space of the sensor is related to the subject’s motion range and the desired placement domain, such as the front wall of the washroom or the back wall of the bathroom. After the position space was determined, we discretized the sensor position space of a specific object (i.e., the body space of the wearable type and the environmental space of nonwearable type). However, the position placed in the real world is expressed in a continuous space. In a virtual environment, although continuously altering the sensor position becomes possible, applying optimization to a continuous position space in a virtual environment is still challenging. One of the solutions to perform continuous position optimization is to generate the dataset after each time of particle movement in the optimization; with that comes greater computational complexity. Even though the computational cost can be neglected, ensuring that the search particles travel on the surface of the human body in three dimensions still requires careful design.

Although discretization of the position space reduces the exploring space to some extent, it still maintains big advantages, namely: (a) decreasing the complexity and (b) ensuring the regular arrangement of sensors (array). The first has two sides. When reducing complexity, the search space is actually reduced as well. The second point enables discrete optimization to be more practical. Even at the expense of the time cost of performing continuous space optimization, the results are still hard to map into the real world. For example, for a nonwearable case under a given sensor number task, the result of continuous position optimization will ultimately become several dots scattered in space. To map such separated sensor points in the real world is hard and not beautiful. Additionally, for wearable sensors (like IMU), the continuous position space is in 3D on the body surface. It is difficult to map a sensor that determines the precise installation angle. While our discrete scheme is closer to the real situation and considers more practical sensor positions for the human body, the considered locations are not only limited to significant joints on the body but can also be concentrated on any limbs of interest. This is why predefining the position space is necessary. Therefore, in this paper, we mainly focus on discrete sensor position spaces; it is helpful to produce a unified interface for different types of HAR designs, including wearable and nonwearable. What needs to be done before optimization is to define the location space and the degree of discretization (how many possible/considered locations there are in total). This step helps the application system become more versatile and mapped to reality.

### 6.2. Sensor Numbers for HAR

In addition to determining the position space, the sensor number should be specified before applying the optimization scheme. For non-visual sensors, the limited information channel restricts the performance of the HAR system to a certain extent. Generally, for a situation in which the accuracy is not satisfied with one sensor, increasing the sensor number is supposed to be a favorable solution for improving accuracy (as in the results for Case 3). However, the space for improving accuracy by increasing the number of sensors is limited, which can cause an overfitting issue and decrease the accuracy. It is also not realistic to pursue accuracy improvement by raising the sensor number because the sensor’s installment must consider the practical situation. Particularly, for the wearable HAR system, wearing more than four or five sensors would be unacceptable for most people in their daily lives (from an empirical point of view). A balance between the number of sensors used and the accuracy desired should be carefully considered. Our method appears to answer the maximum accuracy with a related position under the given sensor number condition. It is helpful to decide whether the current sensor number is acceptable or to check the number of increased results.

### 6.3. Algorithm Performance and Comparison

**Figure 12:**
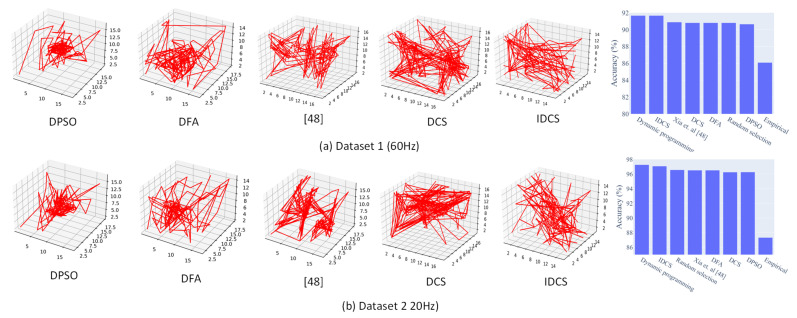
Different heuristic algorithm comparison in Study 1.

The optimizer can be utilized with either a virtual dataset or a real dataset to investigate optimal sensor placement. In Study 1, Figure 12 shows the searching trajectory during the iteration; the DPSO and DFA easily trap in a local optimum. From the trajectory, the DPSO method, in particular, did not explore more space. The performance of PSO is limited to the initial particle distribution position to a large extent. In the method of [14], despite it improving the PSO to avoid the algorithm being trapped into the local optimum, the design of this method is divided into two stages and still needs more iteration times to find the global best solution. Thus, under the lower iteration time requirement (such as 200 times in our study), the output from this method cannot outperform the ID-CS. The CS without interval division mapping can test a wider space but cannot find a global one. The reason is that the Levy flight did not adjust. The movement of particles is usually larger. All the comparisons demonstrated the high performance of the developed ID-CS method—i.e., the closest to the global results and computational time was significantly decreased (e.g., from $C_{17}^{3}$ = 680 to 200).

**Figure 13:**
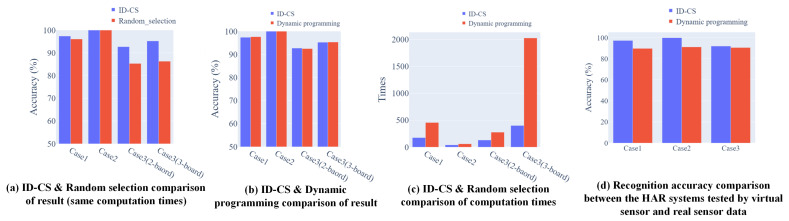
The optimization method comparison in terms of accuracy and computation times; (**a**) result comparison—ID-CS vs. random selection under the same computation cost (highest accuracy reached by ID-CS); (**b**) result comparison—ID-CS vs. dynamic programming (a few differences between ID-CS and DP); (**c**) computation times comparison—ID-CS vs. dynamic programming (less computation cost than ID-CS); and (**d**) accuracy comparison of virtual sensor vs. real sensor data.)

From Figure 13, our ID-CS based optimizer has the advantage of exploring the optimal solution (with a few differences, average of less than 0.2%). The computational cost is reduced significantly, especially when the computation is large (more than 70%). The proposed ID-CS maintains advantages not only at high accuracy but also at lower computational cost. However, as sensor modality and recognized activity are quite diverse, the advantages of exploiting the proposed optimization with virtual sensors are also slightly different. For example, Case 2 of the experiment enabled the wearable distance sensors to design an exercise HAR system. The difference between the methods is quite small. The reason is that the distance signal variations from each defined position all maintain a relatively significant difference under the desired recognized activity and position space. From the results, applying the proposed optimization seems to lead to less improvement in accuracy. Nevertheless, merely conducting the empirical method or the random selection method cannot determine whether there is a possibility of accuracy improvement under the existing conditions. The necessity of understanding the best accuracy in the current position space is still required.

Additionally, the optimizer can be used with various classifier structures. In our platform, SVM was adopted for HAR. Obviously, the ultimate result is likely to be influenced by various classifier networks. However, the classifier selection is only recognized as a relationship between the sensor positions and produced accuracy. Therefore, under multiple application scenes, diverse classifier networks can be determined, which does not affect the use of the optimizer to find the best positions of sensors.

Hence, the proposed method, which combines the virtual sensor with ID-CS optimization, can be recognized as an augmentation tool. It is able to contribute to the decision-making process in terms of the sensor position issues in a convenient way.

### 6.4. Virtual Sensor Based HAR Design

Generally, purely using the virtual dataset for training has a similar effect to the pure real sensor dataset used. We used the five-fold cross validation on the pure real sensor dataset (which is used for testing purposes in three cases) to obtain the accuracy. The final results are 97.38% (only virtual) vs. 97.96% (only real) for Case 1100% (only virtual ) vs. 100% (only real) for Case 2, and 92.07% (only virtual) vs. 92.71% (only real) for Case 3. This shows the virtual sensor data basically have similar data distribution characteristics compared to real situations.

To facilitate the use of optimization, we combined it with virtual sensor use. The preliminary results demonstrated the effectiveness of utilizing virtual motion to design a more flexible HAR system, considering the sensor position. The accuracy comparison between the virtual sensor tested HAR and the real sensor tested is presented in Figure 13d according to all designed cases from 5.3. The results presented relatively good mapping between the virtual sensory and real sensory. However, the mentioned activities for recognition in the three cases were generally simple and of little difficulty. It is believed that recognizing such simple activities in the real world would reach a higher accuracy. The reason for such an accuracy decrease is the signal’s error between the virtual sensor data and the real sensor data. There are basically two main reasons that cause errors between the two signal domains: the inaccuracy of the MoCap capture model and the replay process for virtual signal generation.

In this paper, we selected Xsens as a motion reconstruction device. As a commercial product, the fidelity of the character’s actions is highest following this approach. Even so, there are still errors that are caused not only by the measurement but also by the motion visualization process. Among the methods used to reconstruct 3D virtual motion, the MoCap-based approach is supposed to outperform others. However, such a method is only suitable for small scale group dataset collection.

Additionally, for virtual acceleration generation, the method flows through the interpolation, second differential, and resample process. Thus, the interpolation process can significantly affect the ultimate resampled virtual data. Due to the input virtual motion being frame-by-frame and having already been sampled by a MoCap device, the noise is able to lead to the low performance interpolation. From the confusion matrix in Figure 11, incorrectly recognized activities with a high false-positive rate are related to a high intensity motion, such as upstairs and downstairs, and the leg stretch and squat. As the motion itself will lead to relatively large movement, the sampling and interpolation are likely to generate the data distribution with more errors compared with real sensor data.

For environmental sensors (such as distance sensors), different virtual body shapes can also influence classifier performance, which is the main reason affecting the error recognition rate. As the avatar’s shape was stable, to improve the accuracy, we applied the DA to increase/decrease the body shape of the employed virtual avatar and enabled the virtual data to be closer to the real situation (i.e., real body shape). In this paper, we simply multiplied the initial avatar’s shape by 0.8 and 1.2 to augment the data. The determination of scale-factor follows the experience value and the ultimate effect only reaches an acceptance level around 90%. For further performance improvement, in addition to an advanced virtual sensor-orientated DA approach, a personalized virtual human model can be generated from a personal RGB video. It is likely to contribute to improving the mapping degree between the virtual and real sensor data.

However, we did not intend to test the performance of virtual sensors as this was not the focus of this paper and the effectiveness of virtual sensors has been demonstrated (particularly the virtual IMU in [8,9]). In our experiment, we argued more in relation to the sensor position’s selection in HAR and exploited the virtual sensor data to reduce the burden of real sensor data collection. Thus, we conducted the validation with a small amount of samples. With more accurate and convenient methods to obtain the virtual motion model, combining sensor selection and virtual sensor data to design a HAR system can be explored.

## 7. Limitations and Future Work

### 7.1. HAR System Trained by Virtual sensory

The human modeling tool was adopted to virtualize human activity in the current system. The effect caused by fewer training datasets cannot be eliminated well via DA. Additionally, the tested samples are still limited by the current method. We may argue that the current process is more suitable for small groups of people, such as families. With few input motions collected and virtual sensor use, the developed system will be flexible for such a small group of people to realize a customized HAR system regarding recognized activity and sensor position. To improve the HAR system design, facilitating a widely used system and expanding the input source is necessary. Relying on a diverse video source and vision-processing network, it is possible to expand the current existing sensor dataset (e.g., [8]). Further improving the performance of the visual model and producing a more realistic virtual motion with less noise will be the focus of the next step.

### 7.2. Expanding on Virtual Sensor Modality

In the real world, multiple types of sensors are used to establish the respective HAR systems in different scenarios. With the development of virtualization of human motion and avatars, more motion data can be transferred into 3D virtual motion, which can inspire the development of more virtual sensor modalities to facilitate more types of HAR system design. We believe that this is a potential area that can tackle the issue of datasets’ lack of nonvisual HAR systems, as well as altering the traditional ML-developing fashion through high-performance vision-processing models. In addition, the virtual sensor signal can also associate with VR/AR application to create more interaction.

### 7.3. Multi-Objective Optimization within Wider Space

As previously discussed, the current design discretizes the searching path under the given position space. To expand the searching space and determine more possible global best solutions, a continuous searching strategy can be used because the searching particles can move smoothly in the virtual 3D environment. The main difficulty is restricting the moving trajectory of related searching particles, particularly for wearable types. As the searching space is supposed to be the whole skin area of the human body, it is extremely irregular. Meanwhile, a highly efficient swarm intelligence optimization algorithm is supposed to maintain “randomness” to enable the particle to get rid of the calculated searching path. Thus, how to ensure that the search particles can accurately walk on the avatar surface (and are not inserted into the body) and do not fly out of the body surface under randomness should be the next focus.

Except for the position space, there are other searching spaces that can be explored to convert the accuracy-targeted optimization to a multi-objective optimization, for example, the user’s preference. As a wearable device, users normally have different preferences at placement selection. It is believed that users will be willing to sacrifice some accuracy to meet their own wearing requirements, such as combining the sensor with their accessories, as long as the accuracy is acceptable. Other possible factors, such as price, size, and weight, can also be combined in a multi-objective optimization. An optimization can answer a “sensor scheme” that is optimal to the requirements of recognition accuracy, wearability, sensor features, and so on. It will be a huge effort to push forward a personalized HAR system design for each individual.

## 8. Conclusions

In this paper, we propose a novel optimization of the ID-CS algorithm to explore the optimal sensor position in a HAR system. The proposed method has been demonstrated effectively to be applied both in real and virtual sensor dataset. To the best of our knowledge, this is the first time the sensor position issue has been considered in the designing period and we believe we have proposed a complete and novel method based on virtual sensors to develop the HAR system in regard to the sensor position. Combined with virtual sensor data, the optimization scheme can be used to determine the best sensor position and acquire the related classifier without collecting real sensor data. We utilized the virtual human motion to develop two types of HAR systems, wearable and nonwearable. Through the experiments, the system has demonstrated effectiveness not only in optimizations with fewer computation costs, but also in the performance of using the virtual classifier to recognize real sensor data. With low computational cost optimization and virtual sensory, it is possible to inspire more interaction between the user and the HAR sensor system via the sensor position as an interface. We envision future work that will concentrate on improving the accuracy of virtual sensors and combining them with more interactive virtual animations to generate more classifiers for practical living.

## Figures and Tables

**Table 1:** Performance comparison of different methods with different sampling rate datasets.

Method	Dataset 1 (60 Hz)		Dataset 2 (20 Hz)	
	Result	Best Solution	Result	Best Solution
DPSO	Left shoulder, Left forearm, Right lower leg	90.65%	Left shoulder, Left forearm, Right upper leg	96.26%
DFA	Right upper arm, Left shoulder, Right upper leg	90.81%	Chest, Left forearm, Right upper leg	96.51%
DCS	Chest, Left upper arm, Left lower leg	90.81%	Waist, Left shoulder, Left forearm	96.25%
**ID-CS**	**Chest**, **Right upper arm**, **Right upper leg**	**91.67%**	**Chest**, **Right shoulder**, **Left forearm**	**97.07%**
Xia et. al [14]	Waist, Right shoulder, Right upper arm	90.89%	Chest, Left forearm, Right upper leg	96.51%
Random selection	Chest, Left upper arm, Left lower leg	90.81%	Right shoulder, Left shoulder, Left hand	96.56%
Empirical	Waist, Right upper leg, Right foot	86.09%	Waist, Right upper leg, Right foot	87.32%
**Dynamic** **programming**	**Chest**, **Right upper arm**, **Right upper leg**	**91.67%**	**Waist**, **Right hand**, **Left hand**	**97.26%**

**Table 2:** Results of Case 1 and Case 2, which are both wearable types. Case 1 is a 3-sensor selection for 5 recognized activities. Case 2 is a 2-sensor selection from a virtual wearable distance sensor.

Case	Recognized Activity	Method	Position	Computation Cost	Result
Case 1	Standing/ Walking/ Running/ Upstairs/ Downstairs	**ID-CS**	**Chest**, **Right shoulder**, **Head**	**175**	**97.38%**
		**Dynamic** **programming**	**Chest**, **Right lower leg**, **Left lower leg**	**455**	**97.62%**
		Random selection	Left shoulder, Right upper arm, Right lower leg	175	96.09%
		Empirical	Waist, Right upper leg, Head	1	95.12%
Case 2	Heel up and down/ Squat/ Hip stretch	**ID-CS**	**Left lower leg-left and** **Left upper leg-back**	**40**	**100%**
		**Dynamic** **programming**	**Left lower leg-left and** **Left upper leg-back**	**60**	**100%**
		Random selection	Right upper leg-back and Right upper leg-front	40	100%
		Empirical	Left lower leg-left and Left upper leg-right	1	98.39%

**Table 3:** Result of different sensor board numbers and position selections from virtual distance sensors.

Number of Sub-Board	Method	Position (Location No.)	Computational Cost	Result
1	Dynamic programming	[0, 1, 2, 3, 4]	24	90.86%
2	**ID-CS**	**[0, 1, 2, 3, 4] &** **[5, 6, 7, 8, 9]**	**130**	**92.07%**
	Random selection	[10, 11, 12, 13, 14]& [6, 16, 26, 36, 46, 56, 66]	130	85.32%
	**Dynamic** **programming**	**[0, 1, 2, 3, 4] &** **[15, 16, 17, 18, 19]**	**276**	**92.49%**
3	**ID-CS**	**[0, 1, 2, 3, 4]&[5, 6, 7, 8, 9]** **&[3, 13, 23, 33, 43, 53, 63]**	**400**	**95.23%**
	Random selection	[6, 16, 26, 36, 46, 56, 66]& [0, 1, 2, 3, 4, 3]& [13, 23, 33, 43, 53, 63]	400	86.29%
	**Dynamic** **programming**	**[0, 1, 2, 3, 4]&[15, 16, 17, 18, 19]** **&[3, 13, 23, 33, 43, 53, 63]**	**2024**	**95.33%**

## Data Availability

Not applicable.

## Abbreviations

The following abbreviations are used in this manuscript:

HAR	Human activity recognition
DCS	Discrete cuckoo searching
DPSO	Discrete particle swarm optimization
DFA	Discrete firefly algorithm
DPSO	Discrete particle swarm optimization
ID-CS	Improved discrete particle swarm optimization
